# Dual-color labeled anti-mucin 1 antibody for imaging of ovarian cancer: A preliminary animal study

**DOI:** 10.3892/ol.2014.2807

**Published:** 2014-12-17

**Authors:** QIONG ZHANG, FAN WANG, YAO-SEN WU, KE-KE ZHANG, YAN LIN, XUE-QIONG ZHU, JIE-QIANG LV, XIAO-SHENG LU, XIAO-LEI ZHANG, YUE HU, YIN-PING HUANG

**Affiliations:** 1 Department of Gynecology and Obstetrics, Second Affiliated Hospital, Wenzhou Medical University, Wenzhou, Zhejiang 325027, P.R. China; 2 Department of Orthopedic Surgery, Second Affiliated Hospital, Wenzhou Medical University, Wenzhou, Zhejiang 325027, P.R. China; 3 Department of Gynecology and Obstetrics, First Affiliated Hospital, Wenzhou Medical University, Wenzhou, Zhejiang 325000, P.R. China

**Keywords:** fluorescence imaging, ovarian cancer, mucin 1 antibody, CD227 antibody, near-infrared fluorescence

## Abstract

To investigate the feasibility of the anti-mucin 1 (anti-MUC1/CD227) antibody in the fluorescent imaging of ovarian cancer, the CD227 antibody and a control IgG antibody were labeled with a near-infrared dye [Cy5.5-N-hydroxysuccinimide (NHS)] and a green dye (fluorescein-NHS). *In vivo* fluorescence images were obtained at 4, 12 and 36 h after injection of the probes into OVCAR3 tumor-bearing mice. The tumor to background ratios were calculated for both probes. *Ex vivo* fluorescence images were obtained following sacrifice at 36 h. After conjugation to Cy5.5 and fluorescein, the dual-color labeled CD227 probe (Ab-FL-Cy5.5) could be visualized by both green and near-infrared fluorescence. Uptake by the tumors was higher for the Ab-FL-Cy5.5 than for the IgG-Cy5.5 probe. All tumors could be visualized by *in vivo* imaging with an acceptable tumor to background ratio. *Ex vivo* studies demonstrated the advantages of using green fluorescence imaging to guide the resection of tumor tissues. These preliminary data indicate that the Ab-FL-Cy5.5 probe is promising for further tumor imaging applications and clinical translation.

## Introduction

Ovarian cancer is one of the most common gynecological cancers and a leading cause of cancer-related deaths in females (1). For example, the estimated number of new ovarian cancer cases and mortalities in the United States in 2012 is 22,280 and 15,500, respectively (1). The prognosis for advanced-stage ovarian cancer remains poor. Cytoreductive surgery is the predominant treatment for the majority of ovarian cancers, and removing the maximum amount of affected tissue may improve the efficacy of other subsequent therapies (2). It is vital to distinguish malignant tumors from nearby normal tissue and/or benign lesions. Various imaging methods, including ultrasound, X-ray, computed tomography (CT), magnetic resonance imaging (MRI) and positron emission tomography (PET) are all used for the detection and preoperative evaluation of tumors, however, achieving a high contrast over nearby normal tissues is challenging using conventional imaging modalities. Therefore, more reliable techniques for assisting with cytoreductive surgery are required. Fluorescence imaging is attractive for superior intraoperative tumor detection, and several clinical studies have explored its applications in clinical practices (3,4). A recent study utilized folate conjugated with fluorescein for specific, intraoperative fluorescence imaging of tumor tissue in patients undergoing an exploratory laparotomy for suspected ovarian cancer (3); this demonstrated the feasibility and potential benefit of this unique imaging method.

Fluorescence imaging is advantageous due to its high-resolution, high sensitivity and low cost (5). The most widely used fluorophores are fluorescein and its derivatives, which have extremely high quantum yields and desirable excitation and visible emission wavelengths (6). However, fluorescein also has limitations, including high tissue absorption, tissue scattering and high inference from autofluorescence (7). Near-infrared (NIR) fluorescent dyes, with an emission wavelength ranging from 650–900 nm, can provide real-time, dynamic images of tumors *in vivo* with high sensitivity (8,9). Tumor-targeted fluorescence imaging for cancer diagnosis and treatment has attracted significant attention, and is on the verge of clinical implementation (10). Many investigational clinical studies involving NIR fluorescence imaging have been reported (11,12). Troyan *et al* developed an NIR fluorescence imaging system for image-guided oncologic surgery (11), and Crane *et al* used indocyanin green to detect the sentinel lymph nodes in patients with cervical cancer (12). Folate receptor-α, vascular endothelial growth factor, epidermal growth factor receptor (EGFR), chemokine receptor 4, and matrix metalloproteinase are the five most prominent targets, which have relatively high expression rates in ovarian cancer, high availability of an antibody or substrate, and are promising for translation to human use (10). Mucin 1 (MUC1) is a transmembrane mucin whose extra cellular domain can serve as a ligand for stromal and endothelial cell adhesion receptor, and its cytoplasmic domain plays a role in the cell migration, invasion and survival (13). MUC1 is often overexpressed in metastatic cancers, and its overexpression has been observed in colon, breast, ovarian, lung and pancreatic cancers. It is also a reliable epithelial marker for ovarian carcinoma cells. Additionally, it is a favorable target for immunotherapy, and a number of therapies targeting MUC1 in patients with advanced disease are being assessed in preclinical development or clinical trials (13,14). Monoclonal antibodies have been widely utilized for targeted therapies and may also be used for tumor imaging (15). The present study aimed to evaluate the feasibility of *in vivo* molecular imaging using fluorescent labeled antibodies against MUC1 for the detection of ovarian cancer. The anti-MUC1 antibody (Mouse Anti-Human CD227 antibody) was labeled with a near-infrared dye [Cy5.5-N-hydroxysuccinimide (NHS)] and a green dye (fluorescein-NHS) and evaluated in OVCAR3 tumor-bearing mice.

## Materials and methods

### Reagents

Cy5.5-NHS was purchased from GE Healthcare (Piscataway, NJ, USA). Mouse Anti-Human CD227 antibody (cat. no. 6378-0150, 0108) was purchased from RayBiotech, Inc. (Norcross, GA, USA). Mouse IgG (cat. no. A7208) and fluorescein-NHS were purchased from Beyotime Institute of Biotechnology (Haimen, China). Other chemicals were of analytical grade or better and were used as purchased from Sigma-Aldrich, St. Louis, MO, USA. The human ovarian carcinoma OVCAR3 cell line was purchased from Shanghai Cell Bank of the Chinese Academy of Science (Shanghai, China). Ethics approval was provided by Wenzhou Medical University (Wydw2014-0134).

### Labeling of the antibody

Conjugation of the dyes to the antibody was performed according to the manufacturer’s instructions. The Cy5.5-NHS and fluorescein-NHS dyes were dissolved in dimethyl sulfoxide at a concentration of 10 μg/μl. The antibodies were transferred to 0.1 M sodium bicarbonate buffer (pH 8.5), typically at a concentration of 1 mg/ml. A molar ratio of 10:1 (dye:antibody) was used. Following reaction in the dark at 4°C for two hours, the reaction mixture was purified using Zeba™ Spin Desalting Columns (7K MWCO; Thermo Fisher Scientific, Waltham, MA, USA) to separate the low-molecular-weight dyes from the dye-conjugated antibody. The antibody and the bound dye contents were determined by measuring UV absorption using a Shimadzu UV-1800 spectrophotometer (Nakagyo-ku, Kyoto, Japan).

### Cell culture

The OVCAR3 cells were cultured in Dulbecco’s modified Eagle medium (DMEM; GIBCO, Carlsbad, CA, USA) supplemented with 10% fetal bovine serum and 0.1 μg/ml penicillin-streptomycin. The cells were expanded in tissue culture dishes and incubated in a humidified atmosphere of 5% CO_2_ at 37°C. The medium was replaced every other day. A confluent monolayer was detached using 0.05% Trypsin-EDTA and 0.01M phosphate buffered saline (PBS; pH 7.4), and dissociated into a single-cell suspension for further cell culture.

### Animals

The animal experiment was approved by Wenzhou Medical University Animal Care and Use Committee (Wenzhou, China). Nude mice of six-eight weeks of age and a mean weight of 20 g were divided randomly into two groups: The Ab-FL-Cy5.5 group (n=3) and the IgG-Cy5.5 group (n=3).Approximately 3×10^6^ cultured OVCAR3 cells were suspended in 50 μl PBS and subcutaneously implanted in the right shoulders of female nude mice. Tumors grew to a size of 0.6–1 cm in four weeks.

### In vivo fluorescence imaging

*In vivo* fluorescence imaging was performed using a Kodak In-Vivo FX Pro Imaging System (Kodak, Woodbridge, CT, USA) and analyzed with Kodak Molecular Imaging Software (Kodak). A filter set (excitation wavelength, 610 nm; emission wavelength, 700 nm) was used for achieving NIR fluorescence *in vivo.* Identical illumination settings were used to obtain all images.

For the experiment, mice were injected via the tail vein with 0.5 nmol of probe. Mice in the Ab-FL-Cy5.5 group were injected with Ab-FL-Cy5.5 probe, and mice in the IgG-Cy5.5 group were injected with IgG-Cy5.5, which was used to evaluate the non-specific binding effects of antibodies. NIR fluorescence images were acquired at 4, 12, and 36 h post injection (p.i.). The mice were subsequently sacrificed at 36 h p.i. The tumor and major organs were dissected for evaluation with *ex vivo* fluorescence imaging.

### Statistical analysis

SPSS software, version 17.0 (SPSS Inc., Chicago, IL, USA) was used for data analysis. Measurement data were analyzed using an independent samples t-test, and results are expressed as the mean ± standard deviation. P<0.05 was considered to indicate a statistically significant difference.

## Results

### Labeling of the antibody

**Figure 1:**
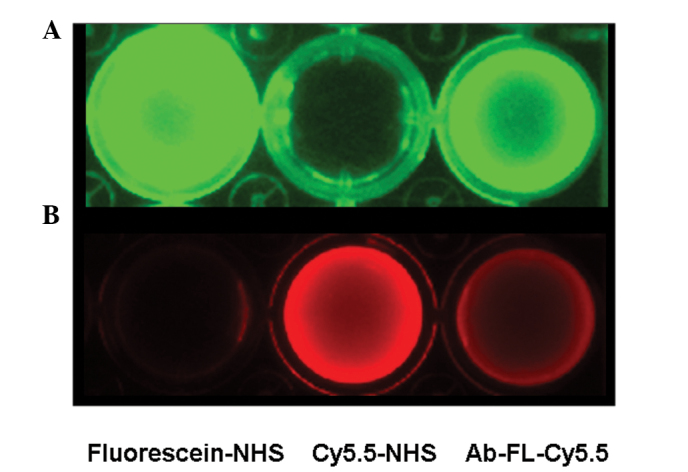
*In vitro* fluorescence images of fluorescein-NHS dye, Cy5.5-NHS dye and the probe Ab-FL-Cy5.5 under different imaging conditions: (A) Excitation wavelength,470 nm; emission wavelength, 530 nm and (B) excitation wavelength, 610 nm; emission wavelength, 700 nm. NHS, N-hydroxysuccinimide.

The average numbers of fluorescein and Cy5.5 molecules per antibody molecule were determined as 2.5 and 2.8, respectively, as calculated by the UV absorptions. *In vitro* fluorescence images of unconjugated fluorescein-NHS and Cy5.5-NHS and the dye-conjugated antibody probe, Ab-FL-Cy5.5, were obtained under different excitation wavelengths (Fig. 1). The dual-labeled antibodies were visible following excitation at wavelengths of 470 and 610nm.

### Fluorescence imaging of OVCAR3 tumor-bearing mice

**Figure 2:**
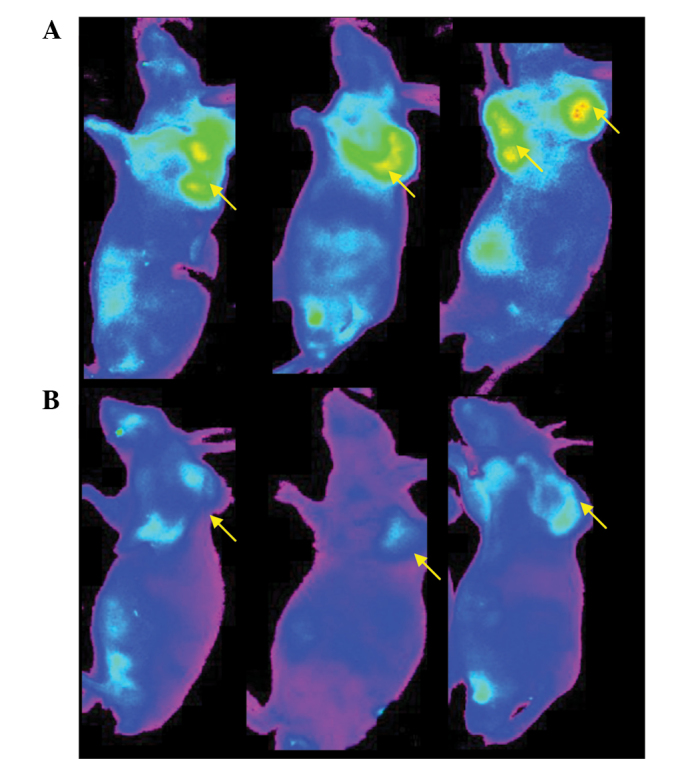
Fluorescence images of tumor-bearing mice 12 h following injection of the (A) Ab-FL-Cy5.5 and (B) IgG-Cy5.5 probes. Arrows indicate the locations of the tumors.

Following injection of Ab-FL-Cy5.5 and IgG-Cy5.5, tumors were visualized in mice from both groups (Fig. 2). The fluorescence signal intensities observed in the tumor regions were higher than those in other regions of the mice.

**Figure 3:**
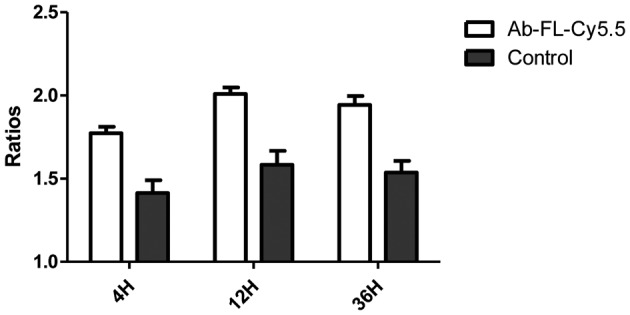
Tumor:background fluorescence ratios at different time points following injection of Ab-FL-Cy5.5 and control (IgG-Cy5.5) in OVCAR3 tumor-bearing mice. OVCAR3, human ovarian carcinoma cell line.

Fluorescence intensity of the mouse’s central back area was set as the background level, and the ratios of tumor to background intensity were calculated. A quantitative analysis of the *in vivo* fluorescence imaging data is represented in Fig. 3. At 4, 12 and 36 h, the tumor:background ratios for Ab-FL-Cy5.5 were 1.77±0.066, 2.01±0.065 and 1.94±0.093, respectively, while the ratios for IgG-Cy5.5 were 1.41±0.13, 1.58±0.14 and 1.53±0.12, respectively. The uptakes of Ab-FL-Cy5.5 were significantly higher compared with those of IgG-Cy5.5 at every time point (P<0.05). The fluorescence intensity reached a plateau at 12 h.

### Ex vivo imaging

**Figure 4:**
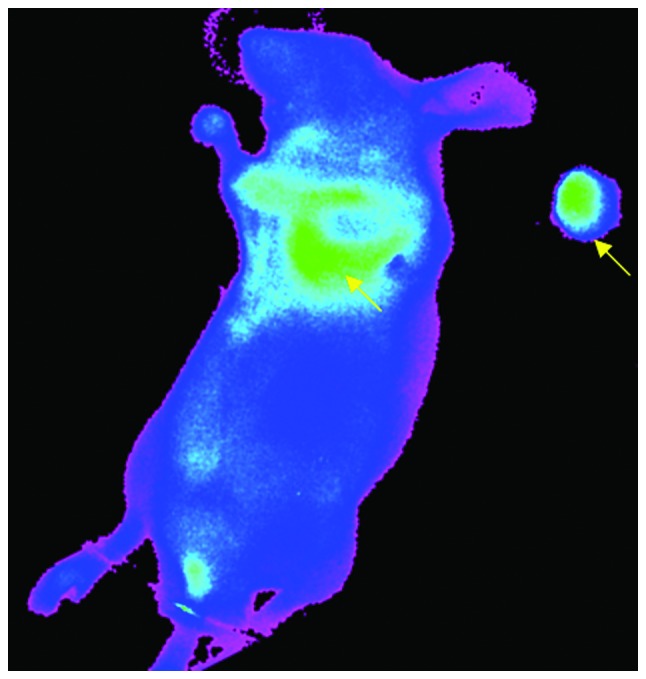
Near-infrared fluorescence imaging of tumor-bearing mouse injected with Ab-FL-Cy5.5, following surgery to remove part of the tumor tissues. Tumor tissues indicated with arrows.

**Figure 5:**
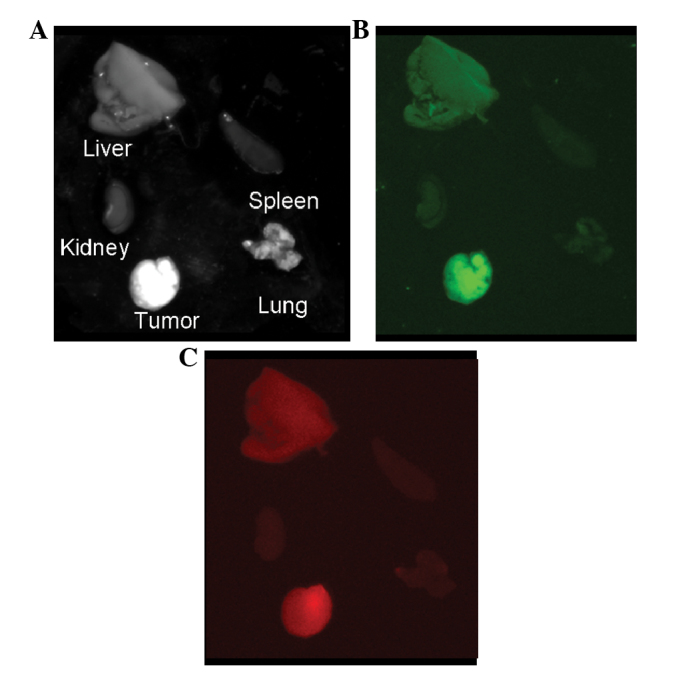
*Ex vivo* images of the main organs of a tumor-bearing mouse 36 h following injection of Ab-FL-Cy5.5: (A) White light, (B) green light (excitation wavelength, 470 nm; emission wavelength, 530 nm) and (C) near-infrared imaging (excitation wavelength, 610 nm; emission wavelength, 700 nm).

Sections of tumor tissue were surgically removed from tumor-bearing mice at 36 h after injection of Ab-FL-Cy5.5. The dissected tumor tissue and the remaining tissues could be clearly visualized by NIR fluorescence imaging (Fig. 4). At 36 h following injection, fluorescence images of the liver, kidney, spleen, lung and tumor were acquired. As shown in Fig. 5, the highest intensity of green and NIR fluorescence was observed in the tumor, and uptake of Ab-FL-Cy5.5 in liver tissue was also observed. Kidney tissue exhibited the lowest signal intensity, while lung and spleen tissue showed a slight uptake of the antibody probe. Signal intensities in the nearby tissues, including healthy ovarian tissue, were observed to be similar to the background intensity.

## Discussion

This preliminary study showed that the Ab-FL-Cy5.5 probe could accumulate in the tumors, while presenting low fluorescence in other organs, which proved its specificity and potential for distinguishing tumor tissue from healthy tissue. These results demonstrate the feasibility of the anti-MUC1 antibody in fluorescent imaging of ovarian cancer and its potential capability to aid surgical procedures. Optimal cytoreductive surgery is important for the treatment of ovarian cancer, and imaging techniques that can effectively differentiate tumor tissue from unaffected tissue are necessary to improve these procedures (16). However, conventional imaging modalities, including CT, MRI and PET, have limitations in the real-time detection of tumor margins during surgery due to the lack of imaging systems for image-guided surgery (11). Many efforts have focussed on the development of new intraoperative imaging techniques to overcome such limitations.

Fluorescence imaging based on specific probes is one of the most promising techniques for intraoperative tumor detection. Visualization of the tumor tissue is achieved via a fluorescence imaging system and tumor-targeted probe labeled with a fluorescent dye, therefore the development of a fluorescent probe with specific targeting ability is vital for imaging-guided surgery. For ovarian cancer, a number of specific targets have been identified, most prominently folate receptor-α, vascular endothelial growth factor, EGFR, chemokine receptor 4, and matrix metalloproteinase (11). In a study by van Dam *et al* (5), folate molecules were conjugated with fluorescein for the successful visualization of tumor tissue in ovarian cancer patients using a real-time intraoperative fluorescence imaging system. Another study evaluated the *in vivo* sensitivity, specificity and diagnostic accuracy of an α(v)β(3)-integrin-targeted NIR fluorescent probe using an intraoperative fluorescence imaging system (16). These two studies demonstrated the feasibility of fluorescence imaging for the intraoperative imaging of ovarian cancer. The development of additional fluorescent probes to specifically target ovarian cancer tissues is of great importance to advance this technique.

In recent years, NIR dye-labeled antibodies have been successfully utilized for the imaging and treatment of tumors, such as malignant gliomas and breast cancer (17). A Cy5.5-labeled anti-EGFR antibody, cetuximab, was used to image head and neck squamous cell carcinoma xenografts *in vivo*, and the results demonstrated the capability of a fluorescently labeled anti-EGFR antibody to be utilized for detecting human tumors in a surgical setting (18). Cy5.5-cetuximab bioconjugate was also evaluated for its potential utility in the detection and guided removal of regional and distant micrometastasis (19). It was found that mice bearing pulmonary metastases displayed remarkable fluorescence across the lung surface after cetuximab-Cy5.5 injection. Panitumumab conjugated with IRDye800 (emission wavelength, 800 nm) has also been used in the imaging of cutaneous head and neck tumors in mice (20). This study demonstrated that panitumumab-IRDye800 had potential to be translated to the clinic for detection and removal of subclinical cutaneous squamous cell carcinoma using Food and Drug Administration-approved imaging hardware.

The present study focussed on the MUC1 antigen to evaluate its potential as a target using an anti-MUC1 antibody conjugated with two dyes: Cy5.5, which is widely used for *in vivo* NIR fluorescence imaging, and a fluorescein derivative, with an emission wavelength in the range of green light (outside the visible range). Numerous fluorescence imaging apparatus already exist for fluorescein derivatives (21). Under the reaction conditions used in the current study, the resulting Ab-FL-Cy5.5 probe had a molecular ratio of antibody:fluorescein:Cy5.5 of 1:2.5:2.8 (approximately five fluorophores per antibody). If too many fluorophores are conjugated to the antibody molecule, self-quenching of the fluorescence may occur, particularly for fluorescein (22).

The tumor to background ratio of the probe in the current study was relatively low, however, the tumor areas could be visualized clearly by *in vivo* and *ex vivo* imaging. Certain studies have demonstrated that IRDye800 exhibits better tumor to background contrast than Cy5.5 (20,23). Use of more specific antibodies may also result in better imaging outcomes.

In conclusion, the preliminary data of this study indicate that MUC1 is a suitable target for ovarian cancer imaging, and anti-MUC1 antibody conjugated with fluorescent dyes is promising for further imaging applications. Moreover, the dual-color labeling strategy was successful and provided more opportunities for the detection of the fluorescence signal. However, the use of more specific antibodies and other dyes, such as IRDye800, may result in improved imaging outcomes; further studies are warranted.
